# Resolving the Nature of the Lowest-Frequency Raman Mode of Liquid Water

**DOI:** 10.1021/acs.jpclett.6c02937

**Published:** 2026-10-05

**Authors:** Florian Pabst, Harald Forbert, Dominik Marx

**Affiliations:** † 19040 SISAA − Scuola Internazionale Superiore di Studi Avanzati, 34136 Trieste, Italy; ‡ Center for Solvation Science ZEMOS, Ruhr-Universität Bochum, 44780 Bochum, Germany; § Lehrstuhl für Theoretische Chemie, Ruhr-Universität Bochum, 44780 Bochum, Germany

## Abstract

The lowest-frequency Raman mode of water, observed through depolarized light scattering or optical Kerr effect techniques, is routinely used to track dynamic changes of interfacial water near ions or biomolecules. Despite this broad importance, its microscopic origin and relation to dielectric relaxation remains debated since decades—with conflicting interpretations even for pure water. To resolve this controversy, we first accurately compute the Raman spectrum down to a few GHz based on ab initio simulations. Second, we introduce detailed decomposition analyses down to the dynamics of nuclei and electrons to reveal that the rotational and translational contributions are equally important. Third, strong negative orientational cross-correlations as well as internal field effects are found to modify the rotational component in very distinct ways. Overall, our analysis down to the electronic structure level provides an unexpectedly complex mechanistic scenario that fully resolves the long-standing debate concerning the lowest-frequency Raman mode of water and its relation to the dielectric spectrum.

## Graphical abstract

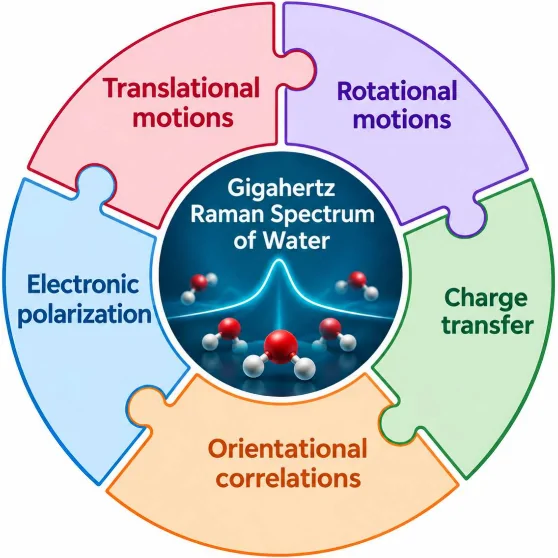

Nearly 100 years ago, shortly after his Nobel Prize-winning discovery of inelastically scattered light from liquids, Raman and his co-worker reported a “nebulosity or wings” in the scattered spectrum close to the incident radiation.^1,2^ Observing that these wings were unpolarized and their intensity scaled with the anisotropy of the molecules, they attributed this part of the spectrum to the rotational motions of molecules in the liquid. However, in the late 1960s, it was discovered that even light scattered by isotropic molecules is not fully polarized as previously expected.^3^ This phenomenon was attributed to the dipole-induced-dipole (DID) mechanism:^4^ The incident light induces a dipole moment in a molecule, which subsequently induces a dipole moment in neighboring molecules, resulting in an effective anisotropy. Since the DID effect depends on the intermolecular distances, it is commonly associated with translational motions.

Despite these findings, the lack of a universally accepted theory of light scattering in liquids has been left unresolved whether rotational or translational motions dominate the low-frequency Raman spectrum. Typically, this part of the spectrum is attributed to rotational motions if the molecule is significantly anisotropic and to translational motions otherwise.^5^ For water, however, both interpretations coexist in the literature. Optical Kerr effect studies, for instance, attribute the lowest-frequency Raman mode to rotational motions,^6^ translational motions,^7^ or both.^8^ Additionally, its appearance in the depolarized light scattering spectrum, at a frequency similar to that of the secondary mode in the dielectric spectrum, is often regarded as evidence of rotational motions.^9,10^ This interpretation stems from the fact that dielectric spectroscopy primarily monitors the rotational motions of molecular electric dipole moments. In this context, the dominant dielectric peak of neat water at approximately 18 GHz is attributed to long-lived hydrogen-bond-mediated orientational cross-correlations between neighboring molecules, which are absent in the Raman spectrum. Understanding the molecular origin of these low-frequency peaks in both Raman and dielectric response of water is key to correctly interpreting experimental spectra as widely used by many communities to investigate slow processes in aqueous solutions, ranging from simple ions and large biomolecules such as proteins to interfaces such as lipid membranes.

Experimental evidence has demonstrated that these cross-correlations explain the discrepancy between dielectric and light scattering spectra for various liquids.^11,12^ For water, in stark contrast, the situation is more complex. A recent ab initio simulation study^13^ revealed that the single-molecule rotational mode in the dielectric spectrum occurs at 30 GHz, which cannot be reconciled with the lowest-frequency Raman mode at around 200 GHz, even accounting for a theoretically expected factor of 3 between the two techniques.^14^

Such a factor of 3 between the peak positions of the two methods is known to be expected if rotational diffusion is the molecular mechanism underlying the dynamics: While the dipole moment is a vectorial quantity, thus corresponding to a correlation function proportional to the first rank Legendre polynomial (∝cos­(θ)), the polarizability tensor is of second rank (∝cos^2^(θ)), explaining the faster decay of the latter. Importantly, very similar peak positions are nevertheless expected in the case of large angular jumps,^14^ for which both correlation functions decay simultaneously upon molecular jumps. In addition, a transition between diffusion and jump mechanism is observed when lowering the temperature in some cases.^15^ Given this background, the observed discrepancy between dielectric and Raman peak positions suggests at first glance that instead of rotational motions, the lowest-frequency Raman mode might be dominated by the DID effect, i.e., by translational motions. Indeed, early force field simulations seemed to support this hypothesis.^16^ However, these simulations were based on anisotropy values obtained in a vacuum, which are now known to be underestimated.^17^

Given these longstanding controversies, our goal is to clarify the yet unresolved microscopic origin of the low-frequency Raman spectrum of pure liquid water at ambient conditions using state-of-the-art explicit ab initio molecular dynamics (AIMD)^18^ simulations; note that we computed for the present investigation converged Raman spectra of ambient water down to a few GHz thus transcending previous explicit AIMD work^19–21^ toward much lower frequencies (note the double-logarithmic scales) while the higher-frequency regime is outside our scope. Given that the electronic structure is accessible allows for the calculation of spectra in close agreement with experiments as has been demonstrated for the dielectric spectrum of water.^13,22^ We will use several decompositions of the total Raman spectrum into contributions of self- and cross-correlations, into DID and intrinsic contributions to the polarizability tensor and we will show how placing all molecules “in a vacuum” helps to disentangle the pure rotational motions at the level of fluctuating nuclei and electrons.

We accurately compute the Raman spectrum in the susceptibility representation via^23,24^

1 $$\chi^{\prime \prime }\left(\omega \right)=\frac{\omega }{6k_{B}TV}\int_{0}^{\infty }e^{-i\omega t}{\left\langle \underset{\kappa \neq \lambda }{\sum }A_{\kappa \lambda }^{e\mathrm{ff}}(0)\;A_{\kappa \lambda }^{e\mathrm{ff}}\left(t\right)\right\rangle }_{cl}\;dt$$

where *T* is the temperature, *V* the volume and ***A*** ^eff^ is the total effective polarizability tensor of the simulation box. We note that what is sometimes called “harmonic quantum correction factor” is embodied in this expression following the Kubo formalism,^24^ which we use to derive (in Section II of the Supporting Information) the classical nonresonant Raman susceptibility eq 1 from the double differential Raman scattering cross section via the quantum fluctuation dissipation theorem. Since we are interested in the depolarized spectrum, only the off-diagonal elements κ ≠ λ are used. Here, ***A*** ^eff^ is calculated from the effective molecular polarizability tensors as $A^{e\mathrm{ff}}=\overset{N}{\underset{i=1}{\sum }}\alpha_{i}^{e\mathrm{ff}}$ , which in turn are obtained from the dipole moments induced by an applied external electric field *E*_0_,

2 $$\mu_{i}^{ind}=\mu_{i}^{E}-\mu_{i}^{0}=\alpha_{i}^{e\mathrm{ff}}E_{0}$$

as explained in the Supporting Information.

The dipole moments are calculated from maximally localized Wannier functions^18,25^ computed from available AIMD trajectories.^13^ We refer to these polarizability tensors as *effective*, following earlier terminology,^26^ because they represent the response observed externally under the influence of an external field. As such, they incorporate both the intrinsic polarizabilities of the molecules and the effects of internal fields^27^ as well as the DID interactions. Consequently, this Raman spectrum is directly comparable to experimental depolarized light scattering data^10^ as shown in Figure 1a) without adjustments. It is important to note that, in such experiments, the absolute intensity of the spectrum is typically not recorded, although it can be measured in principle.^28^ As a result, the experimental spectrum was vertically shifted on the *y*-axis to align with the calculated spectrum. The agreement between the two is excellent, which provides, together with the similarly accurate dielectric loss in panel b), a solid basis for comprehensive spectral dissections and assignment as follows.

**Figure 1:**
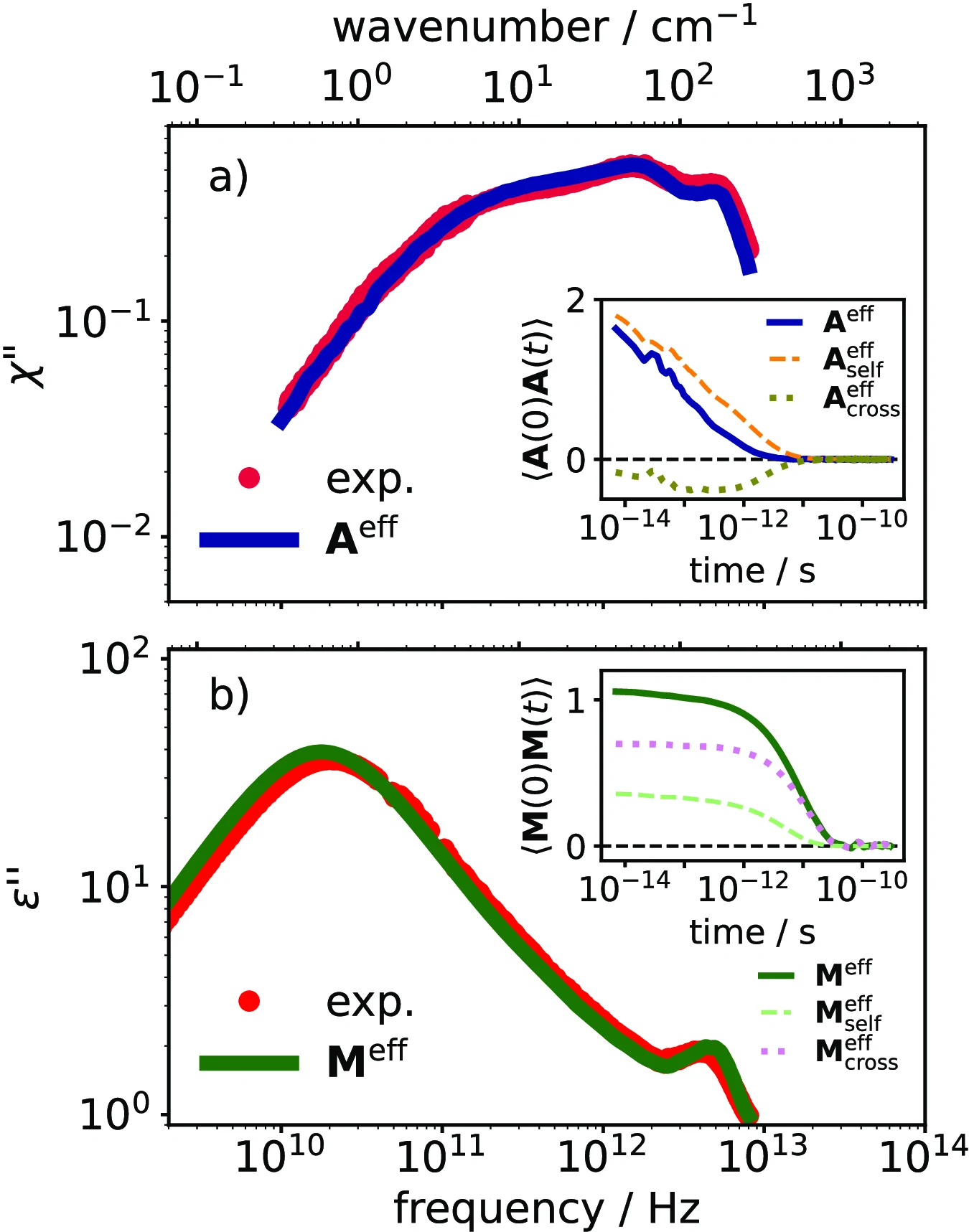
a) Comparison of the experimental depolarized light scattering spectrum^10^ with the one calculated from the AIMD simulations using eq 1. Inset: Total, self part and cross-correlation part of the polarizability correlation function underlying the calculated spectrum in the main panel. b) Same for the dielectric loss spectrum adapted from ref 13 and compared to the experimental dielectric spectrum.^29^

First, we will split the total polarizability tensor ***A*** ^eff^ into contributions from single molecule (self)- and cross-correlations between the molecules. In the inset of Figure 1a), the total, self part and cross-correlation part of the polarizability correlation function

3 $$\left\langle A\left(0\right)\;A\left(t\right)\right\rangle =\underset{self}{\underset{︸}{\left\langle \overset{N}{\underset{i=1}{\sum }}\alpha_{i}\left(0\right)\;\alpha_{i}\left(t\right)\right\rangle }}+\underset{cross}{\underset{︸}{\left\langle \overset{N}{\underset{i=1}{\sum }}\alpha_{i}\left(0\right)\overset{N}{\underset{j\neq i}{\sum }}\alpha_{j}\left(t\right)\right\rangle }}$$

are depicted. While the self part is slightly more intense than the total, the cross-correlation is found to be negative throughout. This is surprising since for the dielectric spectrum the cross-correlation part is roughly a factor of 3 more intense than the self part, thus dominating the total dielectric loss; see the inset of Figure 1b). In the main panel, b), the comparison of the experimental dielectric spectrum^29^ with the one calculated^13^ via

4 $$\varepsilon^{\prime \prime }\left(\omega \right)=\frac{\omega }{3\varepsilon_{0}k_{B}TV}\int_{0}^{\infty }\exp\left(-i\omega t\right)\left\langle M\left(t\right)\;M\left(0\right)\right\rangle \;dt$$

where ***M*** is the total dipole moment of the simulation box, demonstrates quantitative agreement between AIMD and experiment also for this observable; note that eq 4 also contains the harmonic quantum correction factor^24^ consistent with the one applied when computing the Raman spectrum.

For the Raman spectrum, the negative cross part leads to the fact that the total correlation function decays on slightly shorter times than the self part. However, the correlation time of the Raman self part of around 1.2 ps can still not be brought into accordance with the correlation time of the dielectric self part of approximately 5.3 ps, heralding distinctly different underlying microscopic relaxation mechanisms.

Based on this observation, we proceed with another decomposition of ***A*** ^eff^ and scrutinize the earlier suggestion that DID effects are dominating the low-frequency Raman spectrum. In order to disentangle DID from the intrinsic polarizability tensor of the molecules in the liquid state (**α** ^eff^ = **α** ^intr,liq^ + **α** ^DID,liq^), we use a method introduced earlier:^30^ The total field change at each molecule is given by

5 $$f_{i}=E_{0}+\overset{N}{\underset{j\neq i}{\sum }}T_{ij}\;\mu_{j}^{ind}=\left(1+\overset{N}{\underset{j\neq i}{\sum }}T_{ij}\alpha_{j}^{e\mathrm{ff}}\right)E_{0}$$

where the dipole interaction tensor is given by $T_{ij}=\left(3r_{ij}r_{ij}^{T}-1{r_{ij}}^{2}\right)/{r_{ij}}^{5}$ , and the intrinsic polarizability tensor of molecule *i* can be obtained from $\alpha_{i}^{intr,liq}=\alpha_{i}^{e\mathrm{ff}}{\left(1+\overset{N}{\underset{j\neq i}{\sum }}T_{ij}\alpha_{j}^{e\mathrm{ff}}\right)}^{-1}$ , since $\alpha_{i}^{intr,liq}f_{i}=\alpha_{i}^{e\mathrm{ff}}E_{0}$ must hold for all possible ***E***_0_.

The spectra calculated by employing either the total intrinsic or the total DID polarizability tensor when evaluating eq 1 are shown in Figure 2. Several observations can be made: First of all, the intensity of the DID contribution is similar to that of the intrinsic polarizability at low frequencies. This clearly shows that neither of the two can be neglected in this frequency range, contrary to earlier claims. In other words: The DID contribution is not at all negligible in this frequency range as sometimes assumed in theoretical models,^31^ nor is the rotational contribution, contrary to early force field simulations.^16^ Furthermore, also the shape of the low-frequency mode is basically identical for both contributions, highlighting their equal share in shaping the total light scattering spectrum at low frequencies. The inset of Figure 2 shows the self and cross parts of the two contributions. Equivalent observations as for the **α** ^eff^ above can be made, namely, that the self parts relax on slightly longer time scales than the total contribution, while the cross parts are negative.

**Figure 2:**
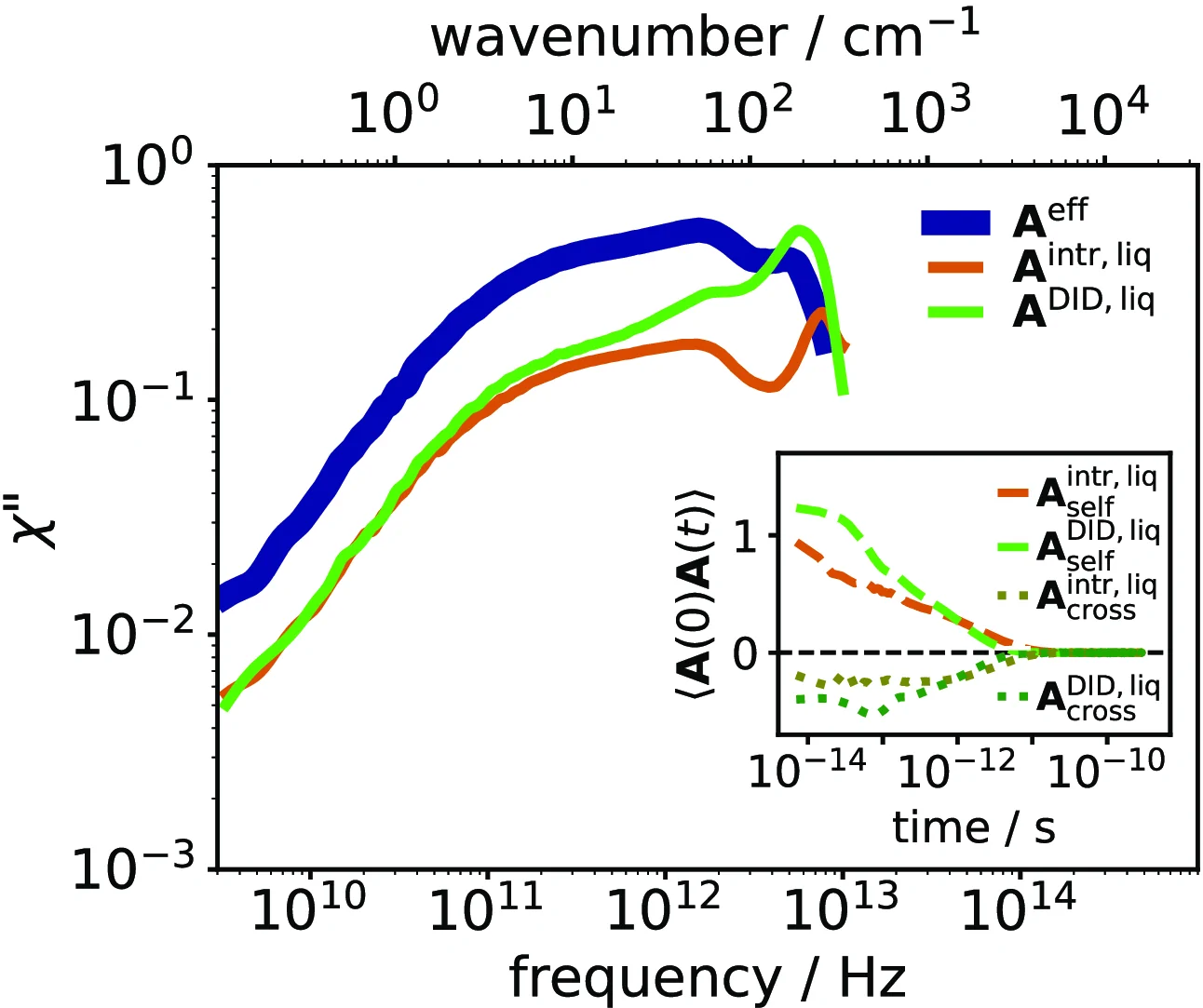
Comparison of the DID and intrinsic contribution to the total Raman spectrum. Spectra are calculated by inserting the respective polarizability tensor as indicated in the legend into eq 1. See also text. Inset: Different contributions to the polarizability correlation functions underlying the spectra in the main panel.

Now, one could expect the self part of the intrinsic polarizability to reflect the single molecule rotational motion, and thus to be in agreement with the dielectric self part. The peak of the lowest mode in the $A_{self}^{intr,liq}$ spectrum is approximately at 70 GHz, which is in line with the single-molecule rotational mode in the dielectric spectrum at 30 GHz after accounting for a theoretical possible factor up to three.^14^ In this context, it must be pointed out that the intrinsic polarizability does not necessarily reflect only the rotational motions. This is because of internal field effects, i.e., surrounding molecules altering the electronic configuration of the molecule under consideration, which render the molecular polarizability tensor different from the one the molecule would have if placed in a vacuum, even for the case of total absence of DID effects. Since the surrounding molecules are continuously moving, this alteration of the electronic structure is a dynamic effect, superimposing the genuine single-molecule rotational motion of the molecule under consideration.

In order to single out the pure rotational contribution, which is not obtainable from a decomposition of ***A*** ^eff^, we conceived the following route: Each single molecule is taken out of its liquid configuration and placed in a vacuum where, maintaining its exact atomic coordinates, its polarizability tensor is recalculated, yielding **α** ^intr,vac^. We introduce this approach to liquid-state Raman spectroscopy to eliminate all effects of the surrounding molecules on the reference molecule under consideration regarding exclusively the electronic structure, while preserving all atomic dynamics along the very same AIMD trajectory in the liquid state. Then, the DID contribution can be calculated to first order in molecular interactions, which has been shown to be sufficient for water,^17^ as follows

6 $$\alpha_{i}^{DID,vac}=\overset{N}{\underset{j\neq i}{\sum }}\alpha_{i}^{intr,vac}T_{ij}\alpha_{j}^{intr,vac},$$

Note that this (approximate) approach is similar but not equivalent to the way Raman spectra have been calculated from force field molecular dynamics simulations. There, however, the polarizability tensor of a water molecule, obtained either from gas-phase experiments^16^ or quantum chemical calculations,^17^ has been fixed to the atomic frame in the simulations. In this way, intramolecular atomic motions are disregarded, which is not the case in our approach. Still, it has to be emphasized that also our procedure is not expected to yield results comparable to experimental data; it is just meant to serve as a rigorous reference to the full spectrum in the liquid state in order to probe internal field effects.

The resulting spectra are shown in Figure 3, where also the purely translational contribution ***A*** ^IDID,vac^ to the DID spectrum is included. It is calculated^32^ by replacing the full molecular polarizability tensors in eq 6 by one-third of their trace, $Tr\alpha_{i}^{intr,vac}/3$ , thus excluding the rotational contributions to $\alpha_{i}^{DID,vac}$ , while keeping only the isotropic (I) part. It can be seen in Figure 3 that ***A*** ^DID,vac^ and ***A*** ^IDID,vac^ are basically identical, which quantitatively confirms the common notion that the DID contribution reflects translational motions.

**Figure 3:**
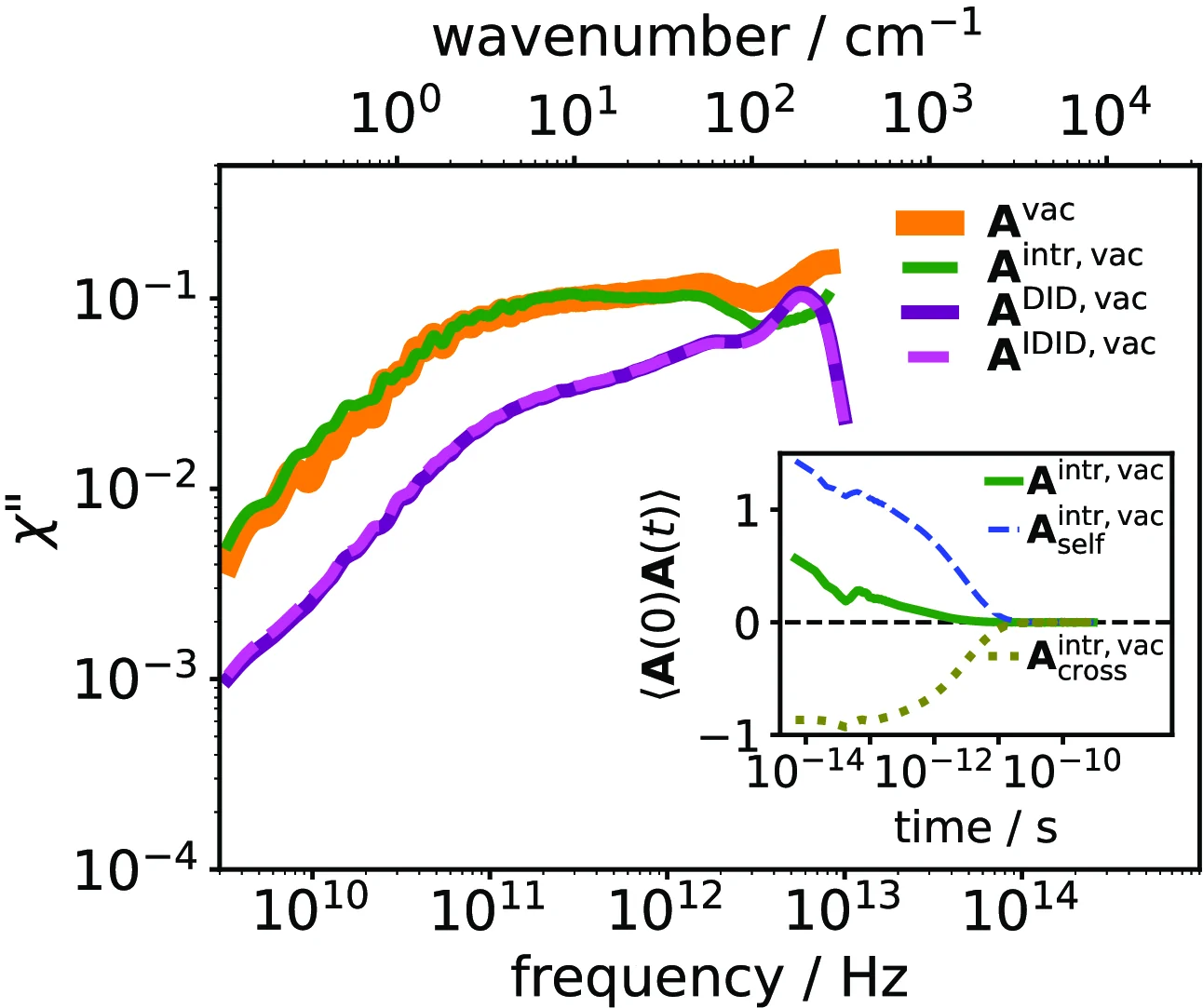
Comparison of the DID, IDID and intrinsic contribution to the total Raman spectrum for the calculations “in a vacuum”. See text. Spectra are calculated by inserting the respective polarizability tensor as indicated in the legend into eq 1. Inset: Total, self-and cross contributions to the intrinsic correlation function.

When comparing the ***A*** ^DID,vac^ to ***A*** ^intr,vac^ spectra, the following observations can be made: The DID contribution is approximately a factor of 4 weaker in intensity than the intrinsic contribution at low frequencies. When comparing to Figure 2, this highlights the fact that the influence of the internal field can markedly influence the weighting of the two contributions.

Now it is interesting to focus on the ***A*** ^intr,vac^ part, as it does not contain local field effects, but solely reflects the rotational motions of the polarizability tensor. In the inset of Figure 3, the splitting into self- and cross-correlation contributions is shown, which are both distinctly different from the total $\left\langle A^{intr,vac}\left(0\right)\;A^{intr,vac}\left(t\right)\right\rangle$ , with the cross-correlation part being strongly negative. Moreover, the correlation decays on a notably longer time scale in the case of the self and cross part as compared to the total. In fact, theoretical considerations predict that the time scale of the total rotational correlation function τ_t_ is shorter than the one of the single molecule τ_s_ in the case of negative orientational cross-correlations.^23^

After having disentangled the single molecule rotation contribution to the light scattering spectrum, we can compare it directly to the dielectric spectrum. To this end, we compute the dielectric loss via eq 4 in two different ways: First, in the liquid state,^13^ i.e., taking all internal field effects into consideration via the maximally localized Wannier centers. The dipole moment calculated in this way is named ***M*** ^eff^ in analogy to the corresponding polarizability tensor. Second, the dipole moment “in a vacuum” ***M*** ^vac^ is calculated in analogy to the polarizability tensor “in a vacuum” as explained above. The comparison of the different dielectric spectra to the corresponding Raman spectra is shown in Figure 4.

**Figure 4:**
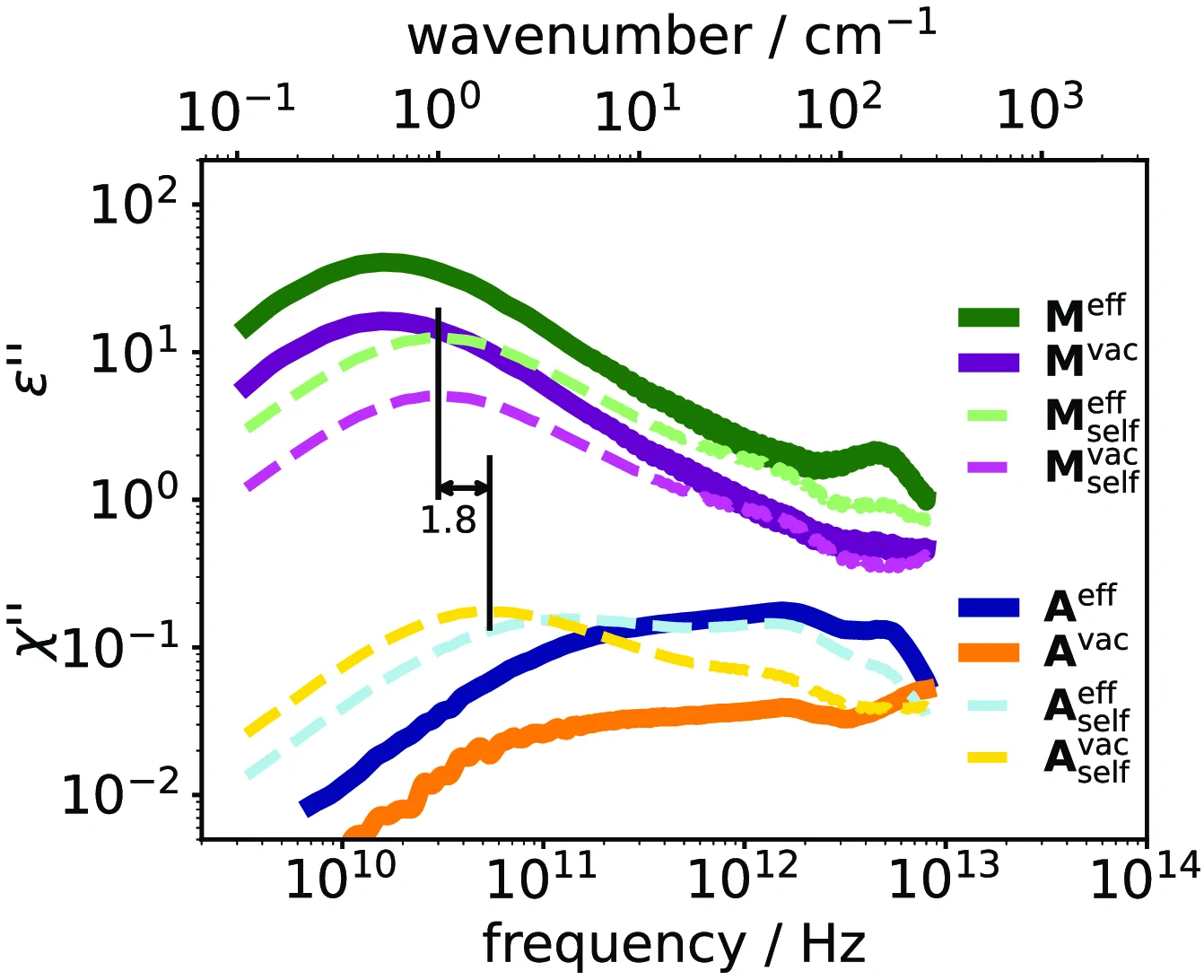
Comparison of dielectric and Raman spectra, calculated by inserting the respective dipole moment into eq 4 or polarizability tensor into eq 1 as indicated in the legend. See text.

One can see that the main difference between the dielectric spectra calculated via these two routes is just the intensity. This is true for the total as well as the self-correlation part of the spectrum. Most importantly, the position of the low-frequency peak does not change in going from the liquid calculations to the “in a vacuum” ones. This means that the internal field just alters the apparent magnitude of the dipole moment, which is well understood.^33^

In contrast, the situation is distinctly different for the light scattering spectrum. Here, the spectra calculated from ***A*** ^eff^ and ***A*** ^vac^ are different not only in intensity but also in shape, due to a different weighting of the individual contributions. Especially the position of the low-frequency peak in the self part of the two spectra is quite different. Notably, it can be seen that the “in a vacuum” self part of the Raman spectrum, which is mainly due to single molecule rotation, is only a factor of 1.8 higher in frequency than the single molecule rotation in the dielectric spectrum. This is now in accordance with simple considerations based on the different ranks of Legendre polynomials involved in the dipole and polarizability correlation functions as reviewed in the introduction. For liquid water at ambient conditions, the observed factor of 1.8 versus 3 thus implies significant deviations from rotational diffusion, i.e., small to medium size angular jumps, in good agreement with the current picture of H-bond dynamics in water in terms of the molecular jump mechanism of water reorientation.^34,35^

Finally, we use our detailed spectral decompositions to derive an intuitive physical picture of the key mechanistic findings to explain the lowest-frequency Raman response of liquid water and its stark differences to dielectric relaxation. We show in Figure 5 a snapshot of some reference water molecule and its four nearest neighbors: For all molecules, the dipole moment vector is represented as a black arrow, while the three eigenvectors of the polarizability tensor, ordered by decreasing magnitude of their corresponding eigenvalues, are shown in orange, green, and blue. The large and positive cross-correlation contribution to the dielectric spectrum, as well as the small and negative cross-contribution to the Raman spectrum, can be rationalized by the mutual orientation of the dipole vectors and the polarizability tensors, respectively, as follows. On the one hand, a preferred local parallel alignment of the dipole vectors can be observed, often quantified by the Kirkwood factor *g*_K_,^36^ eq 7a, whose value is noticeably larger than unity for liquid water (namely *g*_K_ = 2.96(7) computed from the Wannier dipoles along our trajectories), thereby signaling a tendency toward parallel dipole alignment (whereas *g*_K_ = 1 corresponds to uncorrelated random dipole orientations). On the other hand, we obtain for the orientational parameter of the polarizability tensor, *g*_2_, calculated in analogy to *g*_K_ via eq 7b,

7a $$g_{K}=\frac{{∥\overset{N}{\underset{i=1}{\sum }}\mu_{i}∥}^{2}}{\overset{N}{\underset{i=1}{\sum }}{∥\mu_{i}∥}^{2}}$$

7b $$g_{2}=\frac{{{∥\overset{N}{\underset{i=1}{\sum }}\Delta \alpha_{i}∥}_{F}}^{2}}{\overset{N}{\underset{i=1}{\sum }}{{∥\Delta \alpha_{i}∥}_{F}}^{2}}$$

a value of *g*_2_ = 0.939(3); here $\Delta \alpha_{i}=\alpha_{i}-\frac{1}{3}Tr\left[\alpha_{i}\right]I$ is the traceless part of the polarizability tensor and ${∥...∥}_{F}$ denotes the Frobenius norm. We find the *g*_2_ value to be slightly below unity, thus corresponding to weak anticorrelation in agreement with the negative cross-correlation function shown in the inset of Figure 1a (while the dipolar cross-correlations are positive throughout; see inset of panel b).

**Figure 5:**
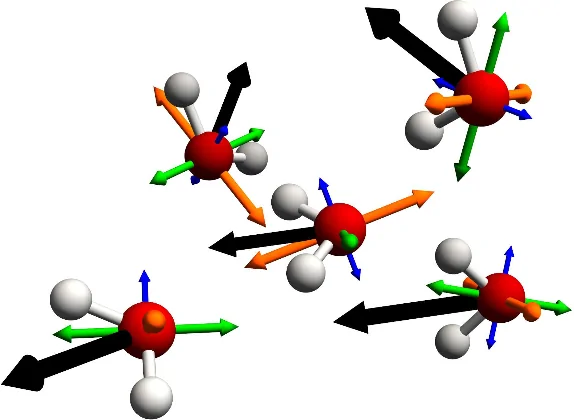
Snapshot of a reference water molecule and its four nearest neighbors. Black arrows are the corresponding dipole vectors while orange, green and blue arrows are the three eigenvectors of the polarizability tensor ordered by decreasing magnitude of their corresponding eigenvalue.

According to this analysis, the mutual orientations of the polarizability tensor axes in Figure 5 are visibly more random (since *g*_2_ ≈ 1) than those of the dipole vectors (where *g*_K_ ≫ 1). Additionally, the effective molecular dipole vectors are preferentially aligned with the bisector of the intramolecular H–O–H angles, as a result of the well-known tetrahedral arrangement of the two covalent O–H bonds and the two lone electron pairs, and therefore perfectly track the rotational motions of the water molecules. In stark contrast, the axes of the polarizability tensors are not as pinned to the moving molecular frame but rather depend crucially on the fluctuating electronic interactions with the dynamical neighboring molecules via the DID and internal field mechanisms as demonstrated above. Our schematic picture visualizes the mechanistic underpinnings of the complex response due to the dynamics of nuclei and electrons that leads to the lowest-frequency Raman mode of liquid water and also explains its key difference compared to dielectric loss.

In summary, supported by the excellent agreement of the computed total low-frequency Raman spectrum compared to experiment, we decompose it into various contributions which unfold an astonishingly complex behavior, including distinct differences from dielectric loss spectra. Both spectroscopic methods are widely used to investigate slow molecular processes in the H-bonded network of interfacial water, for instance close to biomolecules or at membrane surfaces, which critically hinges on a correct molecular understanding of these complementary spectra. Upon introducing systematic dissections of the total response down to the dynamics of nuclei and electrons, we unveil that translational motions, contributing via the dipole-induced-dipole effect, are equally important as the contribution from the intrinsic polarizability, which contains rotational motions and internal field effects. The pure total rotational contribution, on the other hand, is shifted to higher frequencies as compared to the isolated single molecule rotation, due to negative orientational cross-correlations in conjunction with internal field effects. The latter alter the electronic structure of molecules embedded in the liquid as a result of electronic polarization and charge transfer effects compared to isolated molecules. The comparison of the Raman to the dielectric spectrum reveals a rotational jump mechanism as well as that the internal field effect on dielectric loss is much simpler than on Raman response.

Overall, our study provides a comprehensive explanation for the microscopic origin of water’s lowest-frequency Raman mode, which finally settles the case. Despite the intuitive physical picture we provide, the unfolded full mechanistic picture is unexpectedly intricate, which might be the origin of the long-standing controversy. Having understood the pure solvent, we expect our molecular insights to be highly relevant for correctly interpreting the Raman spectra of complex aqueous solutions, as customarily used to investigate how the dynamics of interfacial water are influenced by the presence of ions, biomolecules, or surfaces.^7,17,37,38^ We anticipate that the weighting of the different contributions to the spectrum as identified here might change in the presence of solutes, such as ions and (bio)­molecules, which opens interesting avenues for future research.

## Supplementary Material



## Notes

Details of the simulations and computations as well as the derivation of eq 1 (PDF)
