# Supplementary material for: Resolving the Nature of the Lowest-Frequency Raman Mode of Liquid Water
Source: J Phys Chem Lett. 2026 Oct 5;17(41):11816–21. doi: 10.1021/acs.jpclett.6c02937 (PMC13679086; doi:10.1021/acs.jpclett.6c02937)
Supplement: Supplementary file 1 [file jz6c02937_si_001.pdf]

Supporting Information

Resolving the Nature of the Lowest-Frequency Raman Mode of Liquid Water

Florian Pabst*

SISSA – Scuola Internazionale Superiore di Studi Avanzati, 34136 Trieste, Italy

Harald Forbert

Center for Solvation Science ZEMOS, Ruhr-Universität Bochum, 44780 Bochum, Germany

Dominik Marx

Lehrstuhl für Theoretische Chemie, Ruhr-Universität Bochum, 44780 Bochum, Germany

I. SIMULATION AND COMPUTATIONAL DETAILS

All calculations performed in this work are based on 300 ps long AIMD trajectories from Ref. [S1], using 128 water molecules at 300 K and the experimental density at 1 bar. We stress that it has been carefully checked [S1] that this number of molecules is sufficient for obtaining converged results of the low frequency modes. A subset of 40 trajectories were used here and the calculations of the time-correlation functions underlying the presented spectra were performed with a time step of 7 fs, as opposed to the time step of 1 fs of Ref. [S1], to reduce computational costs related to computing the polarizability tensor along the trajectories. It was checked that the larger time step does not influence the spectra in the frequency range under consideration. To this end, we computed the spectrum for one trajectory on a 1 fs interval and compare it on the left hand side of Fig. S1 to the full spectrum over 40 trajectories on a 7 fs interval. Additionally, we show on the right hand side of Fig. S1 that the spectrum obtained from 40 trajectories is well converged at the lowest frequencies under consideration by comparing four spectra computed over 10 trajectories each.

In order to obtain the position of the Wannier centers with an applied electric field, needed for calculating the polarizability tensors of the water molecules, DFT calculations were performed with CP2K [S2, S3], using the exact same settings as were used to produce the trajectories even though some are irrelevant in this case. This encompasses the use of the RPBE functional [S4] in conjunction with the two-body terms of the D3 dispersion correction with zero damping [S5], as well as the TZV2P basis set [S6], Goedecker-Teter-Hutter pseudopotentials [S7, S8] and a plane wave density cutoff of 500 Ry. We mention in passing that the dispersion-corrected RPBE-D3 density functional has been shown to perfectly reproduce the dielectric loss spectrum of liquid water down to a few GHz [S1]. In addition, it has been broadly used to compute low-frequency vibrational spectra of aqueous solutions involving various solute species as well as water at extreme conditions including nanoconfined and interfacial water – as validated in direct comparison to experimental spectra demonstrated in joint computational-experimental work such as Refs. [S9–S15].

Four different DFT calculations are needed per time step, one without electric field, and three with an electric field applied in x, y and z direction, respectively. A field strength of 5×10^{-3} a.u. was chosen, which is strong enough to reliably obtain shifts of the position of the Wannier centers well above numerical noise, but which still lies well within the linear response regime. From the position of the atoms and the Wannier function centers, the molecular dipole moments were calculated as usual from

$$\boldsymbol{\mu}^{\mathrm{H_2O}} = \sum_{i=1}^{2} e\,\boldsymbol{x}_i^{\mathrm{H}} + 6e\,\boldsymbol{x}^{\mathrm{O}} - \sum_{j=1}^{4} 2e\,\boldsymbol{x}_j^{\mathrm{WFC}} \quad . \tag{S1}$$

In combination with Eq. (2) of the main text, the effective molecular polarizability tensors $\boldsymbol{\alpha}_i^{\mathrm{eff}}$ are obtained.

For the calculation of the spectra "in vacuum", the atomic positions of each individual water molecule were used to calculate the electric dipole moment and the polarizability tensor of the isolated molecule employing the coupled perturbed self-consistent field approach implemented in the ORCA code [S16, S17]. Equivalent settings to the ones

* fpabst@sissa.it

used in CP2K as described above were used, in particular the RPBE functional with D3zero dispersion correction and the def2-TZVPP basis set.

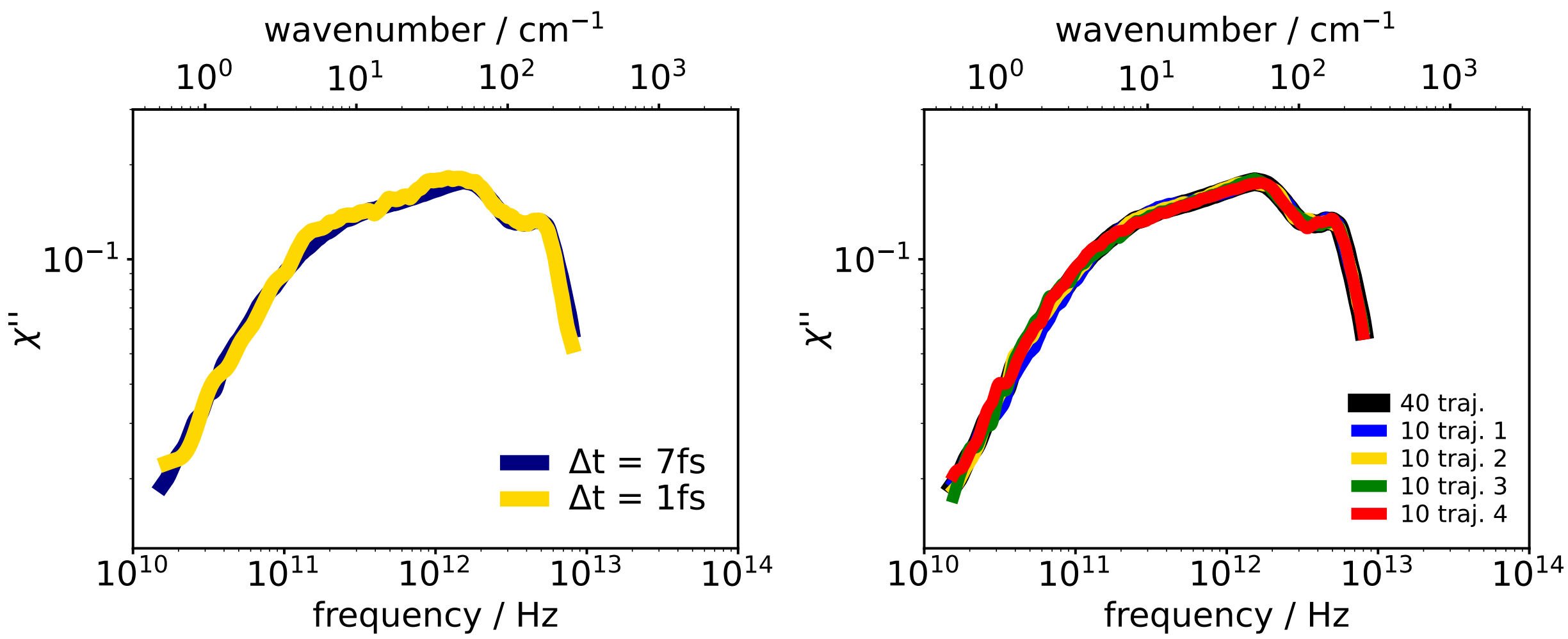

FIG. S1. Left: Comparison of Raman spectra calculated with a time step of 1 fs (1 trajectory) and 7 fs (40 trajectories), respectively. Right: Comparison of 4 Raman spectra calculated from 10 different trajectories each to the full spectrum calculated from 40 trajectories.

II. DERIVATION OF THE CLASSICAL RAMAN SUSCEPTIBILITY

Here, we are going to derive Eq. (1) of the main text by first obtaining the Kubo–transformed quantum susceptibility from the double differential Raman scattering cross section, based on which the classical susceptibility is readily obtained by replacing the Kubo correlation function by its classical counterpart, following our earlier approach [S18]. This method leads to the consistent frequency-dependent prefactor of spectra in the classical limit, called "harmonic quantum correction factor" in the older literature, and has been shown repeatedly to provide excellent quantitative agreement of vibrational spectra with experimental benchmarks, see Figure 2 of Ref. [S1] for instance.

We start with the formula of the quantum fluctuation dissipation theorem for the in non-resonant Raman scattering [S19, S20] relevant polarizability tensor operator $\hat{\boldsymbol{A}}^{\text{eff}}$,

$$2\hbar \frac{1}{1-e^{-\beta\hbar\omega}} \tilde{\chi}''(\omega) = \int_{-\infty}^{\infty} e^{-i\omega t} \left\langle \left(\boldsymbol{\epsilon}^0 \cdot \hat{\boldsymbol{A}}^{\text{eff}}(0) \cdot \boldsymbol{\epsilon}^s\right) \left(\boldsymbol{\epsilon}^0 \cdot \hat{\boldsymbol{A}}^{\text{eff}}(t) \cdot \boldsymbol{\epsilon}^s\right) \right\rangle \mathrm{d}t \,, \tag{S2}$$

where $\boldsymbol{\epsilon}^0$ and $\boldsymbol{\epsilon}^s$ are unit vectors of polarization direction of the incident and scattered light, respectively. If we replace the correlation function by its canonical (Kubo-transformed) counterpart

$$\left\langle \left(\boldsymbol{\epsilon}^0 \cdot \hat{\boldsymbol{A}}^{\text{eff}}(0) \cdot \boldsymbol{\epsilon}^s\right) \left(\boldsymbol{\epsilon}^0 \cdot \hat{\boldsymbol{A}}^{\text{eff}}(t) \cdot \boldsymbol{\epsilon}^s\right) \right\rangle_{\text{Kubo}} = \frac{1}{\beta} \int_0^{\beta} \left\langle \left(\boldsymbol{\epsilon}^0 \cdot \hat{\boldsymbol{A}}^{\text{eff}}(0) \cdot \boldsymbol{\epsilon}^s\right) \left(\boldsymbol{\epsilon}^0 \cdot \hat{\boldsymbol{A}}^{\text{eff}}(t+i\hbar\lambda) \cdot \boldsymbol{\epsilon}^s\right) \right\rangle \mathrm{d}\lambda \,, \tag{S3}$$

we can simplify Eq.(S2) to

$$\tilde{\chi}''(\omega) = \frac{\beta\omega}{2} \int_{-\infty}^{\infty} e^{-i\omega t} \left\langle \left(\boldsymbol{\epsilon}^0 \cdot \hat{\boldsymbol{A}}^{\text{eff}}(0) \cdot \boldsymbol{\epsilon}^s\right) \left(\boldsymbol{\epsilon}^0 \cdot \hat{\boldsymbol{A}}^{\text{eff}}(t) \cdot \boldsymbol{\epsilon}^s\right) \right\rangle_{\text{Kubo}} \mathrm{d}t \,, \tag{S4}$$

since any operator $\hat{B}$ satisfies the Kubo-transform relation [S20]

$$\int_{-\infty}^{\infty} e^{-i\omega t} \left\langle \hat{B}(0)\hat{B}(t) \right\rangle_{\text{Kubo}} \mathrm{d}t = \frac{1}{\beta\hbar\omega} \left(1 - e^{-\beta\hbar\omega}\right) \int_{-\infty}^{\infty} e^{-i\omega t} \left\langle \hat{B}(0)\hat{B}(t) \right\rangle \mathrm{d}t \,. \tag{S5}$$

For isotropic liquids in the case of orthogonal polarization vectors one can spherically average over all possible orthogonal pairs $\boldsymbol{\epsilon}^0, \boldsymbol{\epsilon}^s$ [S20],

$$\tilde{\chi}''_\perp(\omega) = \frac{\beta\omega}{2} \int_{-\infty}^{\infty} e^{-i\omega t} \left\langle \frac{1}{10} \operatorname{Tr}\left(\hat{\boldsymbol{\beta}}(0) \cdot \hat{\boldsymbol{\beta}}(t)\right) \right\rangle_{\text{Kubo}} \mathrm{d}t \; , \tag{S6}$$

where $\hat{\boldsymbol{\beta}} = \hat{\boldsymbol{A}}^{\text{eff}} - \mathbf{1} \operatorname{Tr} \hat{\boldsymbol{A}}^{\text{eff}}/3$ is the traceless part of the polarization tensor operator. Since the diagonal parts of $\hat{\boldsymbol{\beta}}$ are small compared to the corresponding elements of $\hat{\boldsymbol{A}}^{\text{eff}}$ they can still be contaminated numerically by the large trace. Therefore only the off-diagonal parts of $\hat{\boldsymbol{A}}^{\text{eff}}$ are used and thus instead of full spherical averaging only the average over the different sets of perpendicular axes are done here,

$$\tilde{\chi}''_\perp(\omega) = \frac{\beta\omega}{2} \int_{-\infty}^{\infty} e^{-i\omega t} \left\langle \frac{1}{6} \sum_{\kappa \neq \lambda} \hat{A}^{\text{eff}}_{\kappa\lambda}(0) \hat{A}^{\text{eff}}_{\kappa\lambda}(t) \right\rangle_{\text{Kubo}} \mathrm{d}t \; . \tag{S7}$$

Normalizing $\tilde{\chi}''_\perp$ by the volume V and noting that the Kubo–transformed correlation function is symmetric in time the factor of one half can be dropped by integrating only over the positive range of t. Finally, the corresponding exact Kubo–transformed correlation function is approximated by its classical version,

$$\chi''_\perp(\omega) = \frac{\omega}{6 k_{\text{B}} T V} \int_{0}^{\infty} e^{-i\omega t} \left\langle \sum_{\kappa \neq \lambda} A^{\text{eff}}_{\kappa\lambda}(0) A^{\text{eff}}_{\kappa\lambda}(t) \right\rangle_{\text{cl}} \mathrm{d}t \; , \tag{S8}$$

which is Eq. (1) of the main text.

[S1] C. Hölzl, H. Forbert, and D. Marx, Dielectric Relaxation of Water: Assessing the Impact of Localized Modes, Translational Diffusion, and Collective Dynamics, Phys. Chem. Chem. Phys. **23**, 20875 (2021).

[S2] J. Hutter, M. Iannuzzi, F. Schiffmann, and J. VandeVondele, CP2K: Atomistic Simulations of Condensed Matter Systems, WIREs Comput. Mol. Sci. **4**, 15 (2014).

[S3] T. D. Kühne, M. Iannuzzi, M. Del Ben, V. V. Rybkin, P. Seewald, F. Stein, T. Laino, R. Z. Khaliullin, O. Schütt, F. Schiffmann, *et al.*, CP2K: An Electronic Structure and Molecular Dynamics Software Package-Quickstep: Efficient and Accurate Electronic Structure Calculations, J. Chem. Phys. **152** (2020).

[S4] B. Hammer, L. B. Hansen, and J. K. Nørskov, Improved Adsorption Energetics Within Density-Functional Theory Using Revised Perdew-Burke-Ernzerhof Functionals, Phys. Rev. B **59**, 7413 (1999).

[S5] S. Grimme, S. Ehrlich, and L. Goerigk, Effect of the Damping Function in Dispersion Corrected Density Functional Theory, J. Comput. Chem. **32**, 1456 (2011).

[S6] J. VandeVondele and J. Hutter, Gaussian Basis Sets for Accurate Calculations on Molecular Systems in Gas and Condensed Phases, J. Chem. Phys. **127** (2007).

[S7] S. Goedecker, M. Teter, and J. Hutter, Separable Dual-Space Gaussian Pseudopotentials, Phys. Rev. B **54**, 1703 (1996).

[S8] C. Hartwigsen, S. Gœdecker, and J. Hutter, Relativistic Separable Dual-Space Gaussian Pseudopotentials from H to Rn, Phys. Rev. B **58**, 3641 (1998).

[S9] A. Esser, H. Forbert, F. Sebastiani, G. Schwaab, M. Havenith, and D. Marx, Hydrophilic Solvation Dominates the Terahertz Fingerprint of Amino Acids in Water, J. Phys. Chem. B **122**, 1453 (2018).

[S10] H. Vondracek, S. Imoto, L. Knake, G. Schwaab, D. Marx, and M. Havenith, Hydrogen-Bonding in Liquid Water at Multikilobar Pressures, J. Phys. Chem. B **123**, 7748 (2019).

[S11] F. Sebastiani, C. Y. Ma, S. Funke, A. Bäumer, D. Decka, C. Hoberg, A. Esser, H. Forbert, G. Schwaab, D. Marx, and M. Havenith, Probing Local Electrostatics of Glycine in Aqueous Solution by THz Spectroscopy, Angew. Chem. Int. Ed. **60**, 3768 (2021).

[S12] I. Kolling, C. Hölzl, S. Imoto, S. R. Alfarano, H. Vondracek, L. Knake, F. Sebastiani, F. Novelli, C. Hoberg, J.-B. Brubach, P. Roy, H. Forbert, G. Schwaab, D. Marx, and M. Havenith, Aqueous TMAO Solution under High Hydrostatic Pressure, Phys. Chem. Chem. Phys. **23**, 11355 (2021).

[S13] S. Ruiz-Barragan, F. Sebastiani, P. Schienbein, J. Abraham, G. Schwaab, R. R. Nair, M. Havenith, and D. Marx, Nanoconfinement Effects on Water in Narrow Graphene-Based Slit Pores as Revealed by THz Spectroscopy, Phys. Chem. Chem. Phys. **24**, 24734 (2022).

[S14] K. Mauelshagen, P. Schienbein, I. Kolling, G. Schwaab, D. Marx, and M. Havenith, Random Encounters Dominate Water-Water Interactions at Supercritical Conditions, Sci. Adv. **11**, eadp8614 (2025).

[S15] T. Tabassum, B. Das, C. Park, S. Ghosh, D. Naujoks, S. M. Piontek, A. Ludwig, D. Marx, and P. B. Petersen, Hydrophobic Versus Hydrophilic Nature of the Gold-Water Interface Determined by Fluctuating Local Water Orientation, Phys. Rev. Lett. **135**, 116201 (2025).

[S16] F. Neese, The ORCA Program System, WIREs Comput. Molec. Sci. **2**, 73 (2012).

[S17] F. Neese, Software Update: The ORCA Program System, Version 5.0, WIREs Comput. Molec. Sci. **12**, e1606 (2022).
[S18] R. Ramırez, T. López-Ciudad, P. Kumar P, and D. Marx, Quantum Corrections to Classical Time-Correlation Functions: Hydrogen Bonding and Anharmonic Floppy Modes, J. Chem. Phys. **121**, 3973 (2004).
[S19] R. G. Gordon, Relations Between Raman Spectroscopy and Nuclear Spin Relaxation, J. Chem. Phys. **42**, 3658 (1965).
[S20] D. A. McQuarrie, *Statistical Mechanics* (University Science Books, Sausalito, CA, 2000).
